# From Health to Environment: Exploring the Associations Among Health Status, Health-Related Lifestyle, and Campus Environment in Chinese Universities

**DOI:** 10.3390/healthcare14121667

**Published:** 2026-06-11

**Authors:** Guorui Chen, Bo Zhang, Yicheng Zhang, Kun Song

**Affiliations:** 1 School of Architecture and Art, North China University of Technology, Beijing 100144, China; abaodoc@ncut.edu.cn; 2 School of Architecture, Tianjin University, Tianjin 300072, China; urd@tju.edu.cn

**Keywords:** university students’ health, health-related lifestyle, campus environment, two-stage analysis, China

## Abstract

Background/Objectives: College student health has become a global public health concern, with campus environments serving as critical resources for supporting healthy lifestyles. This study aimed to identify heterogeneous associations between health-related lifestyle parameters and health status among university students, as well as the relationships between these parameters and campus environmental factors. Methods: A two-stage analytical approach was applied to 909 student responses from five Chinese universities. Stage One employed hierarchical regression to identify lifestyle parameters significantly associated with health status. Stage Two used EFA-derived factors and LASSO robustness checks to examine campus environmental factors linked to these key parameters. Results: Six lifestyle parameters were significantly associated with student health: physical exercise frequency, physical exercise duration, active commuting frequency, nature contact frequency, healthy diet frequency, and self-rated health literacy. Each parameter exhibited distinct patterns of environmental association. Conclusions: These findings provide empirical evidence for redefining health-promoting campus design through targeted environmental interventions.

## 1. Introduction

The worsening health of college students has become a global public challenge. In China, national surveys show that the physiological function indicators and obesity rates of college students have been deteriorating since 1985 [1]. Now, 21.48% of Chinese college students have different degrees of depression symptoms, and 45.28% of Chinese college students bear the risk of anxiety [2]. The heavy curriculum and complex social pressure faced during the higher education period make it a process with an established trend of damaging health [3], and it may cause a long-term impact [4].

In this situation, “Healthy Campus” has become the priority agenda of higher education institutions (HEI) both globally and in China, including the WHO’s “Health-Promoting University” [5], the American “Healthy Campus” [6], and the Chinese “Healthy Schools” [7]. These programs all position campus HEIs as key population health promotion settings. Unlike medical interventions targeting diagnosed diseases, campus-level health-promoting strategies aim to create supportive environments that enable healthy lifestyles to become routine choices for all students [8]—a preventive approach that aligns closely with the social ecological paradigm in public health [9,10,11].

Promoting a healthy lifestyle is regarded as the core path of healthy campus construction [12]. Modifiable behavioral patterns, such as active commuting (walking and cycling) [13], physical exercise [14], nature contact [15], healthy eating [16], and health education [17], are considered predictors of physical and mental health. The socio-ecological model suggests that individual lifestyle is influenced by multiple factors [11,18], among which the physical environment plays a particularly significant role. For college students, whose daily activities are largely concentrated on campus, the physical environment, including spatial layout [19], green space systems [20], pedestrian networks [21], and facility provision [22], continuously and profoundly shapes both opportunities and behavioral constraints.

Numerous studies have attempted to quantify the associations between the campus environment, student lifestyle, and health outcomes. A wide range of characterization parameters have been employed in these studies to describe the various factors that constitute lifestyle. Horizontally, these parameters encompass different categories, such as active commuting [23], physical exercise [24], nature contact [25], and dietary behaviors. Vertically, they mainly consist of two primary dosage characterization parameters, namely, frequency [26,27] and duration [28,29].

In terms of physical activity, the US Physical Activity Guidelines Advisory Committee, in a report based on hundreds of epidemiological studies, believes that total physical activity duration is associated with a reduced risk of various diseases and mortality [30]. Meanwhile, retrospective studies have reported that most exercise interventions combining duration and frequency have achieved significant results among campus populations [31]. The World Health Organization (WHO) and Chinese health authorities also provide physical activity recommendations combining duration and frequency [32,33]. In terms of natural exposure, most studies generally use duration as a metric by which to measure college students’ level of natural exposure [34]. However, some studies have shown that college students who are regularly exposed to natural environments have better health levels [35,36]. Typical healthy diet scales, such as HEI, AHEI, and DASH, incorporate both total food intake and behavioral frequency [37]. Other studies have used the combined effect of frequency and duration parameters to measure levels of physical activity and nature contact, such as walking distance [38] and metabolic equivalents [39].

Environment–behavior theory and environmental stress theory posit that the physical environment shapes health both directly and indirectly [40]. Based on the mediating role of these parameters, the campus environmental factors related to student health cover multiple dimensions, such as green space accessibility [27], walkability [41,42], sports facilities [43], and spatial quality [44]. These findings have advanced our understanding of how the environment correlates population health through lifestyle factors. The identified environmental factors provide evidence for assessing the health-supportive quality of campus environments and for informing corresponding design strategies.

**Figure 1:**
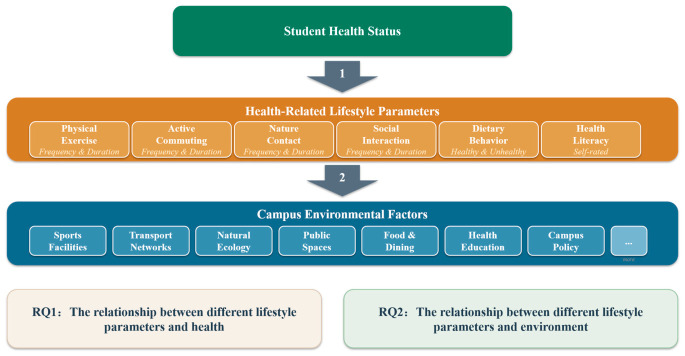
The theoretical framework of this study.

Building on the aforementioned theoretical foundations, this study conceptualizes campus environment, health-related lifestyle, and student health status as an interconnected system (Figure 1). Campus environmental attributes operate through multiple pathways—differentiated by lifestyle parameter and behavioral dosage dimension—to shape health outcomes.

However, the substantial body of existing research presents two issues that require further clarification. First, existing studies often focus on analyzing one or a few specific dimensions of activity parameters. However, a more systematic comparison is needed to determine which lifestyle characterization parameters have a relatively significant impact on health outcomes and to assess the relative importance of different parameters.

Second, given the heterogeneous effects of different lifestyle parameters, the differential roles of environmental factors need further clarification. For example, do patterns of environmental association differ across behavioral parameters?

These more nuanced relational issues are essential for deepening our understanding of how the environment correlates with health, and they directly drive a more precise understanding of the assessment and design of health-promoting campus environments.

This study represents a systematic exploration aimed at addressing the aforementioned issues. A survey on student health status, health-related lifestyle parameters, and campus environmental conditions across five Chinese universities was conducted. A two-stage mixed research and analytical approach was employed to sequentially examine the associations between various lifestyle characterization parameters and student health outcomes, as well as the campus environmental factors associated with these characterization parameters. The heterogeneous health value of lifestyle characterization parameters and the differential effects of related environmental factors were identified. This study further provides evidence for the assessment and design of health-promoting campus environments and establishes a foundation for additional research.

## 2. Method

### 2.1. Study Design

**Figure 2:**
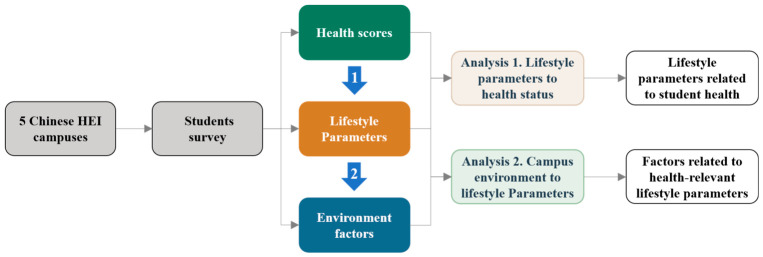
The study design.

This study adopted a cross-sectional survey-based design comprising a two-stage analytical framework. First, five university campuses in China were purposively selected based on their geographic location, campus size, environmental characteristics, and data availability. Second, a structured questionnaire was administered across these campuses to collect information on three domains: campus environmental attributes, health-related lifestyle characterization, and student health outcomes. Third, the associations between health outcomes and lifestyle characterization parameters were examined to identify the parameters significantly associated with student health. Fourth, using these identified lifestyle characterization parameters as dependent variables, the influence of campus environmental factors was further analyzed (Figure 2).

### 2.2. Data

The campus cases in this study are derived from the Beijing–Tianjin–Hebei region of China, which encompasses one of the country’s three core urban agglomerations and serves as both the birthplace of Chinese higher education and a concentration of higher education resources. In 2024, HEIs in this region contributed approximately 12% of the nation’s undergraduate students and 16% of master’s and doctoral students [45], making them illustrative cases that reflect the diversity of Chinese HEIs in terms of location, scale, and spatial configuration. While not a statistically random sample, the purposive selection enhances contextual diversity and analytical generalizability. The selected campus cases satisfied the following conditions: (a) the campus is a closed-campus type, which is the most common form among Chinese HEIs; (b) the campus contains relatively comprehensive facilities and spaces that cover most of the students’ daily needs; (c) the campus has a stable number of new and enrolled students. In the specific selection process, the influence of different campus locations, scales, and spatial configurations was also taken into account to minimize the interference of case-specific characteristics, enhance the representativeness of the data collected, and identify common patterns of influence (Table 1).

The data in this study consist of three parts: student health status, health-related lifestyle, and campus environment.

The first part (Q6–17) assesses student health status, which was measured using the SF-12 scale [46]—a scale developed from the Medical Outcomes Study (MOS) Item Short Form Health Survey.

The second part (Q18–29) covers 12 parameters describing health-related lifestyle, including the daily duration and weekly frequency of physical exercise, active commuting (walking/cycling), nature contact, and social interaction; the frequency of healthy and unhealthy dietary consumption; and students’ self-rated health literacy. Each parameter was presented as a positively worded behavioral statement (e.g., “I frequently engage in physical exercise” for exercise frequency). Respondents indicated their level of agreement with each statement on a five-point Likert scale (1 = strongly disagree; 5 = strongly agree), yielding a subjective self-report of the corresponding lifestyle parameter.

The third part (Q30–74) concerns the characterization of the campus environment. Through a literature screening and structured evaluation process based on the PRISMA framework, this study identified 12 environmental assessment tools for healthy campuses or health-promoting universities from three databases: Web of Science, Scopus, and PubMed. From these tools, 557 specific indicators were extracted. Following systematic organization and consolidation, 45 core campus environmental topics were ultimately derived. Among these, 34 topics describe different physical environmental characteristics of the campus, which were categorized into two aspects: physical performance and material resources. The remaining 11 topics describe non-physical conditions that support the physical environment or facilitate its use, classified as health services and health atmosphere. All topics were expressed as positively worded statements, and respondents rated their level of agreement based on their own perceptions (Table 2). A five-point Likert scale (1 = strongly disagree; 5 = strongly agree) was used for data collection in the second and third parts to ensure consistency in the rating scales.

From September 2024 to January 2025, an online questionnaire was distributed across the five campuses. Respondents accessed it via QR codes at high-traffic areas (academic, living, and landscape zones, as well as a mixed-use zone for TJU-B). Sampling continued on each campus until feedback approached approximately 1% of its student population. Incomplete (with missing answers) and unreliable (e.g., with many identical choices) questionnaires, as well as those collected during the winter break, were removed. The consistency of responses was checked using repeated items in the questionnaire. From the total of 1731 submissions, 909 valid questionnaires from undergraduate and master’s students were finally obtained (Table 3).

### 2.3. Analysis

(1) Variable Preparation

The independent and dependent variables in this study were obtained through a questionnaire survey. For categorical variables, gender (male/female) and accommodation type (living on campus/off campus) were converted into binary dummy variables (0/1) based on their semantic meanings. Four campus dummy variables were created with BUEA (1) as the reference and subsequently included in the regression models. Cronbach’s α for all scale items is 0.793.

(2) Stage One

To examine associations between health-related lifestyle dimensions and student health status, we conducted hierarchical (block-wise) regression with SF-12 health score as the dependent variable. Block 1 (baseline) included demographic variables (gender, grade, accommodation, length of campus residence) and the four campus dummies to control for campus-level fixed effects. Then, conceptually grouped lifestyle parameters were added in subsequent blocks:

Block 2 added physical activity parameters (frequency and duration of physical exercise and active commuting).

Block 3 added nature contact and social interaction parameters (frequency and duration of nature contact and social interaction).

Block 4 added dietary and literacy parameters (frequency of healthy eating, unhealthy eating, health education participation, and self-rated health literacy).

All variables within each block were entered simultaneously. The significant variables (*p* < 0.05) were retained as key lifestyle parameters for Stage Two.

(3) Stage Two

Stage Two examined campus environmental factors associated with the five key lifestyle parameters identified in Stage One.

The 45 environmental items were subjected to exploratory factor analysis (EFA) with oblique rotation (Oblimin). Factors with eigenvalues greater than 1 were retained, yielding 12 composite indexes. Factor scores were saved for subsequent analyses. The internal consistency of each index was checked (Cronbach’s α > 0.70). Factor scores were saved for subsequent analyses (Appendix C).

For each key lifestyle parameter, 6 hierarchical regression models were constructed for the key lifestyle parameters. In each model, Block 1 contained demographic variables and campus dummies (as in Stage One), which controlled for potential confounding effects of individual background and campus-level differences. Block 2 added the 12 EFA-derived indexes as independent variables.

To assess the robustness of the environmental correlates, LASSO regression with 5-fold cross-validation was performed for each key lifestyle parameter, using the 12 indexes (together with demographic variables and campus dummies) as predictors. The penalty parameter λ was selected using the one-standard-error rule (λ.1se). The LASSO results were then compared with the hierarchical regression findings to evaluate the stability of associations across methods.

## 3. Results

### 3.1. Lifestyle Parameters and Health Status

Table 4 presents the hierarchical regression results for self-rated health status (*n* = 903). In Block 1 (campus and demographic controls), female gender (β = −0.143, *p* < 0.01) and higher grade (β = −0.086, *p* < 0.01) were associated with lower health scores (Adj-R^2^ = 0.023).

Adding physical activity in Block 2 substantially improved fit (ΔR^2^ = 0.231, ΔF(4, 890) = 69.647, *p* < 0.001; Adj-R^2^ = 0.252). Physical exercise frequency (β = 0.179), exercise duration (β = 0.307), and active commuting frequency (β = 0.130) were all positively associated with health (all *p* < 0.001).

Block 3 added nature contact and social interaction parameters (ΔR^2^ = 0.010, ΔF(4, 886) = 3.071, *p* = 0.016; Adj-R^2^ = 0.259). Only nature contact frequency showed a significant positive association (β = 0.147, *p* < 0.01).

Block 4 (full model) further included dietary behavior and health literacy (ΔR^2^ = 0.029, ΔF(4, 882) = 9.138, *p* < 0.001; Adj-R^2^ = 0.285). Healthy diet frequency (β = 0.126, *p* < 0.001) and self-rated health literacy (β = 0.140, *p* = 0.001) emerged as significant correlates. Exercise frequency (β = 0.115, *p* = 0.012), exercise duration (β = 0.162, *p* = 0.002), active commuting frequency (β = 0.127, *p* < 0.001), and nature contact frequency (β = 0.113, *p* = 0.023) remained significant.

Consequently, six lifestyle parameters were retained for Stage Two: physical exercise frequency, physical exercise duration, active commuting frequency, nature contact frequency, healthy diet frequency, and self-rated health literacy.

### 3.2. Campus Environment and Lifestyle Parameters

#### 3.2.1. Exploratory Factor Analysis of Campus Environmental Items

EFA of the 45 campus environmental items yielded a 12-factor solution explaining 68.11% of the total variance. The KMO measure was 0.816, and Bartlett’s test of sphericity was significant (χ^2^ = 15,985.98, df = 990, *p* < 0.001), confirming adequate factorability. Table 4 presents the rotated factor loadings and communalities. All 45 items loaded saliently (≥0.40) on a single factor without substantial cross-loadings (all secondary loadings < |0.40|), indicating a clear factor structure. Communality values ranged from 0.523 (Q44, road ancillary facilities) to 0.828 (Q40, public transit connection).

The 12 factors were labeled as follows:

(1) Pedestrian Infrastructure (F1, 8.01% variance): six items concerning road maintenance, width, connectivity, ancillary facilities, safety, and accessible facilities (loadings: 0.718–0.814);
(2) Outdoor Environmental Quality (F2, 7.27%): five items covering outdoor microclimate, air quality, acoustic environment, lighting, and sanitation (loadings: 0.745–0.850);
(3) Landscape Environment (F3, 7.05%): five items on landscape accessibility, diversity, maintenance, ancillary facilities, and environmental safety (loadings: 0.739–0.820);
(4) Healthy Food Environment (F4, 6.08%): four items regarding the quantity, accessibility, promotion, and supply of healthy food (loadings: 0.813–0.830);
(5) Indoor Environmental Quality (F5, 6.05%): four items on indoor lighting, thermal comfort, acoustic environment, and sanitation (loadings: 0.806–0.827);
(6) Daily Service & Social Accessibility (F6, 5.68%): four items on commercial/service facility diversity, commercial accessibility, support facility accessibility, and social space accessibility (loadings: 0.776–0.827);
(7) Sports Facilities (F7, 4.95%): three items on the adequacy, variety, and accessibility of fitness facilities (loadings: 0.849–0.860);
(8) Campus Activities & Academic Support (F8, 4.87%): three items on activity variety, class schedule reasonableness, and academic support (loadings: 0.842–0.857);
(9) Health Policy & Promotion (F9, 4.85%): three items on health-related policies, education opportunities, and information dissemination (loadings: 0.824–0.866);
(10) Medical & Psychological Services (F10, 4.85%): three items on psychological counseling, medical services, and mental health services (loadings: 0.831–0.864);
(11) Indoor Spatial Quality (F11, 4.77%): three items on indoor natural elements, corridor/staircase convenience, and ergonomic furniture (loadings: 0.813–0.859);
(12) Land Use & Transit Connectivity (F12, 3.68%): two items on land utilization efficiency and public transit connection (loadings: 0.891–0.908).

Composite scores for each factor were computed as the mean of their constituent items and used as independent variables in the subsequent regression analyses. Internal consistency for the 12 factors is reported in Table 5.

#### 3.2.2. Hierarchical Regression and LASSO Validation

To examine the associations between campus environmental factors and the six significant lifestyle parameters identified in Stage One, a hierarchical (blocked) multiple regression was conducted for each outcome. In Block 1, campus fixed effects (four campus dummy variables, with Campus 1 as the reference) and demographic controls (gender, grade, accommodation type, and length of campus life) were entered. In Block 2, the twelve environmental factor scores derived from the EFA were simultaneously forced into the model. Standardized regression coefficients (β), exact *p*-values, and model diagnostics (Adj-R^2^, F, ΔR^2^, ΔF) are reported. Variance inflation factors (VIF) for all environmental factors in the full model ranged from 1.062 to 1.181, indicating no severe multicollinearity.

To assess the robustness of variable selection against multicollinearity and overfitting, LASSO (Least Absolute Shrinkage and Selection Operator) regression was performed for each lifestyle outcome as a sensitivity analysis. A cross-validated lambda (λ) was determined for each outcome, and variables with non-zero coefficients at the λ.1se were considered retained. Variables retained by LASSO were compared with the OLS hierarchical regression results to evaluate selection stability.

(1) Physical activities

Table 6 presents the hierarchical regression and LASSO validation results for three physical activity parameters.

For physical exercise frequency, Block 2 significantly improved model fit (ΔR^2^ = 0.192, ΔF(12, 882) = 17.830, *p* < 0.001; Adj-R^2^ = 0.192). Six environmental factors showed significant positive associations: F7 Sports Facilities (β = 0.324, *p* < 0.01), F2 Outdoor Environmental Quality (β = 0.277, *p* < 0.01), F1 Pedestrian Infrastructure (β = 0.211, *p* < 0.01), F12 Land Use & Transit Connectivity (β = 0.130, *p* < 0.01), F3 Landscape Environment (β = 0.095, *p* < 0.01), and F6 Daily Service & Social Accessibility (β = 0.079, *p* < 0.05). LASSO regression (λ.1se = 0.15) retained F7 Sports Facilities and F2 Outdoor Environmental Quality, confirming these as the most robust predictors of exercise frequency.

For physical exercise duration, Block 2 also significantly improved fit (ΔR^2^ = 0.132, ΔF(12, 882) = 11.336, *p* < 0.001; Adj-R^2^ = 0.123). Four factors reached significance: F8 Campus Activities & Academic Support (β = 0.311, *p* < 0.01), F7 Sports Facilities (β = 0.166, *p* < 0.01), F2 Outdoor Environmental Quality (β = 0.163, *p* < 0.01), and F9 Health Policy & Promotion (β = 0.133, *p* < 0.01). LASSO (λ.1se = 0.17) retained only F8 Campus Activities & Academic Support, indicating that campus activity variety and reasonable course scheduling are the most distinctive environmental drivers of exercise duration.

For active commuting frequency, Block 2 yielded a substantial improvement (ΔR^2^ = 0.181, ΔF(12, 882) = 16.333, *p* < 0.001; Adj-R^2^ = 0.166). Seven factors were significantly associated: F1 Pedestrian Infrastructure (β = 0.298, *p* < 0.01), F2 Outdoor Environmental Quality (β = 0.288, *p* < 0.01), F3 Landscape Environment (β = 0.269, *p* < 0.01), F12 Land Use & Transit Connectivity (β = 0.231, *p* < 0.01), F8 Campus Activities & Academic Support (β = 0.120, *p* < 0.01), F5 Indoor Environmental Quality (β = 0.065, *p* < 0.05), and F10 Medical & Psychological Services (β = 0.063, *p* < 0.05). LASSO (λ.1se = 0.08) retained five factors: F1, F2, F3, F8, and F12, indicating broad environmental association with active commuting behavior.

(2) Nature contact and communication behavior

Block 2 significantly improved model fit (ΔR^2^ = 0.115, ΔF(12, 882) = 9.668, *p* < 0.001; Adj-R^2^ = 0.104). Six environmental factors showed significant positive associations: F3 Landscape Environment (β = 0.238, *p* < 0.01), F11 Indoor Spatial Quality (β = 0.211, *p* < 0.01), F5 Indoor Environmental Quality (β = 0.190, *p* < 0.01), F1 Pedestrian Infrastructure (β = 0.134, *p* < 0.01), F8 Campus Activities & Academic Support (β = 0.129, *p* < 0.01), and F2 Outdoor Environmental Quality (β = 0.096, *p* < 0.01). LASSO (λ.1se = 0.12) retained only F3 Landscape Environment, confirming that landscape accessibility, diversity, and maintenance are the core environmental drivers of students’ frequency of nature contact (Table 7).

(3) Diet and literacy

Table 8 presents the results for healthy diet frequency and self-rated health literacy. For healthy diet frequency, Block 2 produced the largest incremental improvement among all outcomes (ΔR^2^ = 0.310, ΔF(12, 882) = 33.711, *p* < 0.001; Adj-R^2^ = 0.308). Three factors were significantly associated: F4 Healthy Food Environment showed the strongest effect (β = 0.573, *p* < 0.01), followed by F9 Health Policy & Promotion (β = 0.102, *p* < 0.01) and F2 Outdoor Environmental Quality (β = 0.062, *p* < 0.05). LASSO (λ.1se = 0.33) retained only F4 Healthy Food Environment, confirming that the availability, accessibility, and promotion of healthy food options dominate students’ dietary behavior.

For self-rated health literacy, Block 2 significantly improved fit (ΔR^2^ = 0.260, ΔF(12, 882) = 26.268, *p* < 0.001; Adj-R^2^ = 0.256). F9 Health Policy & Promotion showed the strongest association (β = 0.491, *p* < 0.01), followed by F10 Medical & Psychological Services (β = 0.279, *p* < 0.01), F4 Healthy Food Environment (β = 0.167, *p* < 0.01), F8 Campus Activities & Academic Support (β = 0.098, *p* < 0.01), F1 Pedestrian Infrastructure (β = 0.071, *p* < 0.05), and F5 Indoor Environmental Quality (β = 0.063, *p* < 0.05). LASSO (λ.1se = 0.14) retained F9 Health Policy & Promotion and F10 Medical & Psychological Services, confirming that institutional health promotion measures and accessible medical services are the most robust environmental predictors of students’ health literacy.

## 4. Discussion

### 4.1. Lifestyle Parameters Related to Student Health Status

Stage One identified six lifestyle parameters significantly associated with health status among the 903 student participants. The hierarchical regression model, controlling for gender and grade level in Block 1, showed a substantial increase in explained variance from an adjusted R^2^ of 0.023 to a final adjusted R^2^ of 0.285. The largest incremental contribution emerged from physical activity parameters (ΔR^2^ = 0.231), followed by dietary and health literacy factors (ΔR^2^ = 0.029), and natural and social contact variables (ΔR^2^ = 0.010). These findings suggest that multiple dimensions of daily lifestyle are linked to self-reported health, with behavioral parameters showing particularly strong associations.

Among the six significant parameters, physical exercise duration demonstrated the strongest association with health status (β = 0.162, *p* = 0.002), followed by physical exercise frequency (β = 0.115, *p* = 0.012). This aligns with existing evidence linking regular physical activity engagement to better health outcomes in student populations [47]. The finding is consistent with dose–response literature linking total weekly physical activity volume to health outcomes [32].

A novel finding was the significant association between active commuting frequency and health status (β = 0.127, *p* < 0.001), even after controlling for structured physical exercise. In contrast, active commuting duration did not reach statistical significance. This suggests that habitual incorporation of active transportation into daily routines, irrespective of individual commuting length, is linked to better health status. Active commuting may serve as an accessible form of incidental physical activity integrated into academic life, offering health-related benefits through both increased energy expenditure and enhanced psychological well-being associated with outdoor movement [48]. The dissociation between commuting frequency and duration echoes a broader pattern: the regularity of engagement appears more closely associated with health status than the length of individual episodes.

Nature contact frequency was significantly associated with health status (β = 0.113, *p* = 0.023), whereas nature contact duration was not statistically significant. This finding suggests that repeated exposure to natural environments, rather than prolonged single episodes, is more closely linked to health benefits. This pattern is consistent with frameworks proposing that frequent nature contact supports restoration and stress reduction through repeated opportunities for attention recovery [29]. The null finding for nature contact duration may reflect diminishing returns in a single nature exposure episode, where brief but regular contact with green spaces is sufficient to produce measurable associations with health perceptions [49,50]. From a practical standpoint, encouraging students to incorporate brief but habitual nature encounters into daily routines may be more beneficial than promoting occasional extended visits.

Healthy diet frequency showed a significant positive association with health status (β = 0.126, *p* < 0.001). This finding is consistent with the well-established link between dietary quality and health outcomes in young adult populations [51]. Students who reported consuming healthy foods more frequently also reported better overall health status, suggesting that dietary patterns are linked to self-assessed well-being beyond the contributions of physical activity and other lifestyle factors [16].

Self-rated health literacy was significantly associated with health status (β = 0.140, *p* = 0.001), representing the strongest cognitive parameter in the model. This finding suggests that students’ confidence in their ability to access, understand, and apply health-related information is linked to their perceived health status. Health literacy may operate through multiple pathways, including more informed decision-making, better adherence to preventive behaviors, and enhanced self-management capacity [52].

### 4.2. Campus Environmental Factors Related to Health-Related Lifestyle Parameters

#### 4.2.1. Physical Exercise: Divergent Environmental Drivers of Frequency and Duration

Physical exercise frequency and duration emerged as environmentally distinguishable constructs. The frequency of exercise (Q18) was most strongly associated with Sports Facilities availability (β = 0.324, *p* < 0.001) and Outdoor Environmental Quality (β = 0.277, *p* < 0.001), followed by Pedestrian Infrastructure (β = 0.211, *p* < 0.001). The LASSO confirmed only Sports Facilities and Outdoor Environmental Quality as robust predictors. These findings suggest that the decision to exercise frequently is linked to the perceived adequacy, diversity, and accessibility of sports facilities, as well as the quality of the outdoor microclimate, air quality, and acoustic environment. This shows that facility provision and outdoor comfort are key correlates of student participation in physical activity.

In contrast, exercise duration (Q19) was predominantly associated with Campus Activities and Academic Support (β = 0.311, *p* < 0.001), with Sports Facilities (β = 0.166, *p* < 0.001) and Outdoor Environmental Quality (β = 0.163, *p* < 0.001) playing secondary roles. Notably, LASSO retained only Campus Activities and Academic Support as robust predictors. This divergence is conceptually important: while facility availability and outdoor conditions appear linked to whether students exercise at all, the amount of time they spend exercising is more closely related to the diversity of organised activities and the reasonableness of course scheduling. This pattern is consistent with the notion that structured campus programs and manageable academic timetables create temporal opportunities for sustained physical activity [53], whereas facility provision alone does not guarantee longer exercise sessions. The differential LASSO selection patterns between Q18 (two factors retained) and Q19 (one factor retained) underscore that frequency and duration are not interchangeable measures and respond to distinct environmental configurations.

#### 4.2.2. Active Commuting: Multidimensional Environmental Support

Active commuting frequency (Q20) demonstrated the broadest environmental associations among all parameters examined, with LASSO retaining five of seven significant factors. Pedestrian Infrastructure (β = 0.298, *p* < 0.001), Outdoor Environmental Quality (β = 0.288, *p* < 0.001), and Landscape Environment (β = 0.269, *p* < 0.001) emerged as the three strongest predictors, followed by Land Use and Transit Connectivity (β = 0.231, *p* < 0.001) and Campus Activities and Academic Support (β = 0.120, *p* < 0.001). The prominence of walkability-related factors—footpath maintenance, connectivity, safety, and landscape amenities—mirrors findings from urban mobility studies showing that pedestrian-oriented infrastructure and esthetically pleasing routes are associated with higher rates of active transport [13,21]. The additional contribution of Land Use and Transit Connectivity points to the importance of efficient spatial organization and public transport integration in supporting walking or cycling as viable commuting options. The inclusion of Campus Activities and Academic Support in the LASSO-selected set further suggests that when campus programming and scheduling accommodate flexible movement patterns, students are more likely to commute actively. Collectively, these results indicate that active commuting is supported by an integrated environmental system spanning infrastructure, outdoor comfort, landscape quality, land use planning, and campus activity design.

#### 4.2.3. Nature Contact: The Centrality of Landscape Environment

Nature contact frequency (Q22) was primarily associated with Landscape Environment (β = 0.238, *p* < 0.001), which was the sole factor retained by LASSO. This finding highlights the pivotal role of landscape accessibility, diversity, maintenance, and perceived safety in facilitating student engagement with natural settings. Although Indoor Spatial Quality (β = 0.211, *p* < 0.001), Indoor Environmental Quality (β = 0.190, *p* < 0.001), Pedestrian Infrastructure, and Campus Activities also showed significant associations in Block 2, their effects did not survive LASSO regularization, suggesting they may operate as secondary or correlated pathways rather than independent drivers. The dominance of Landscape Environment aligns with attention restoration theory and related evidence that the availability and quality of nearby green spaces are linked to more frequent nature contact and associated psychological benefits [20]. From a campus planning perspective, these results imply that investments in landscape quality and accessibility may yield the most direct returns in promoting student interaction with nature.

#### 4.2.4. Diet and Health Literacy: Targeted Environmental Levers

Healthy diet frequency (Q26) was overwhelmingly associated with the Healthy Food Environment (β = 0.573, *p* < 0.001), which was the only factor retained by LASSO. The magnitude of this association—substantially larger than any other parameter–environment pairing in the Stage Two results—indicates that the availability, accessibility, promotion, and consistent supply of healthy food options on campus are tightly linked to students’ dietary patterns. The negligible contributions of Health Policy and Promotion and Outdoor Environmental Quality, though statistically significant in Block 2, did not withstand LASSO shrinkage, reinforcing the primacy of direct food environment interventions. This finding is consistent with prior research demonstrating that food availability and accessibility in educational settings are strongly related to dietary choices among young adults [54].

Self-rated health literacy (Q29) was most strongly associated with Health Policy and Promotion (β = 0.491, *p* < 0.001) and Medical and Psychological Services (β = 0.279, *p* < 0.001), both retained by LASSO. The large coefficient for Health Policy and Promotion points to the critical role of institutional health policies, educational opportunities, and information dissemination in shaping students’ perceived capacity to obtain, process, and understand health-related information. The additional contribution of Medical and Psychological Services suggests that the availability of counseling, medical care, and mental health support is linked to self-assessed health literacy, possibly through repeated exposure to health information during service encounters. The inclusion of Healthy Food Environment (β = 0.167, *p* < 0.001) and Campus Activities and Academic Support (β = 0.098, *p* = 0.001) in the significant but non-retained set further indicates that health literacy may be indirectly supported by environments that embed health-relevant knowledge in everyday campus experiences.

## 5. Limitations

This study has several limitations. First, the cross-sectional design precludes causal inference, and reverse causality cannot be ruled out; healthier students may perceive environments more positively or self-select into supportive settings. Second, all measures were self-reported via a single questionnaire, raising common method bias concerns; objective measures (e.g., accelerometry, GPS) would complement the Likert-based assessments. Third, the five campuses were purposively sampled from one region with imbalanced sample sizes, limiting generalizability despite the use of campus fixed effects. Future research could adopt longitudinal designs, integrate objective measurement tools, expand to more diverse contexts, and explore interactions between frequency and duration parameters as well as the role of socioeconomic status.

## 6. Conclusions

First, six lifestyle parameters were significantly associated with student health: physical exercise frequency, physical exercise duration, active commuting frequency, nature contact frequency, healthy diet frequency, and self-rated health literacy. The patterns of association varied across behaviors: for physical exercise, both frequency and duration showed a significant health link, whereas for active commuting and nature contact, only frequency remained significant. These contrasting patterns underscore the need to distinguish behavioral dosage dimensions when evaluating health relevance.

Second, each of these six parameters was linked to a distinct configuration of campus environmental factors, with LASSO validation confirming the robustness of key associations. Sports Facilities and Outdoor Environmental Quality were the most robust correlates of exercise frequency; Campus Activities and Academic Support was the sole LASSO-retained predictor of exercise duration; Pedestrian Infrastructure and Landscape Environment were the primary correlates of active commuting frequency; Landscape Environment dominated nature contact frequency; Healthy Food Environment overwhelmingly predicted healthy diet frequency; and Health Policy and Promotion, together with Medical and Psychological Services, were the primary correlates of health literacy.

These findings indicate that health-promoting campus design requires targeted environmental interventions rather than generic approaches. Different lifestyle parameters point to different environmental levers: exercise frequency calls for facility provision and outdoor quality, exercise duration for flexible scheduling, nature contact for distributed landscape accessibility, healthy diet for food environment availability, and health literacy for institutional policies and services. Moreover, because frequency and duration are associated with distinct environmental configurations, the same intervention is unlikely to promote both more frequent and longer engagement simultaneously. Future research should build on these findings through longitudinal designs, objective measurements, and expanded samples.

## Figures and Tables

**Table 1:** Information on campus cases.

No.	Campus	Property	Description	General Layout (A. Landscape; B. Living Area; C. Academic Area; D. Mixed-Use Area)
1	Beijing University of Civil Engineering and Architecture (BUEA)	Region: Beijing Built in: 2015 Scale: 62 ha Plot ratio: 0.81 Students: 8000	Intensive suburban campus with small land use, high plot ratio, and relatively complete campus construction	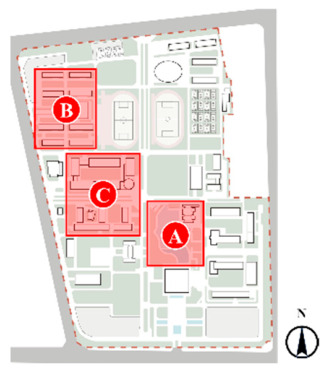
2	North China University of Technology (NCUT)	Region: Beijing Built in: 1958 Scale: 30 ha Plot ratio: 1.33 Students: 16,000	Intensive urban campus, small scale, highly compact land use, high plot ratio, and high building density	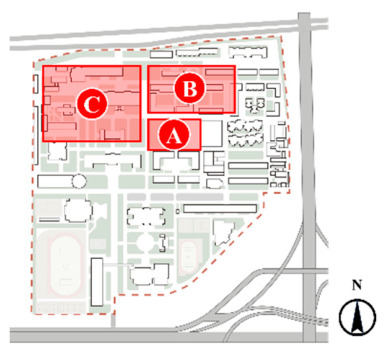
3	Tianjin University—Weijin Rd. Campus (TJU-W)	Region: Tianjin Built in: 1952 Scale: 182 ha Plot ratio: 0.78 Students: 25,000	High density urban campus, long established, large-scale land use, high plot ratio	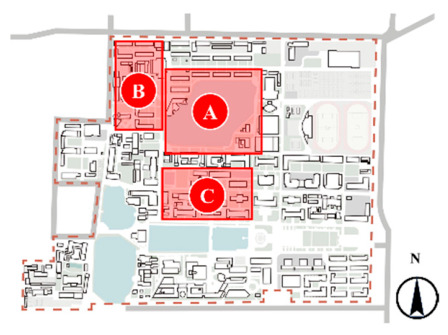
4	Hebei University of Engineering (HUE)	Region: Hebei Built in: 2019 Scale: 273 ha Plot ratio: 0.28 Students: 28,000	Low density suburban campus, with large scale, low plot ratio, and a relatively low proportion of built-up area	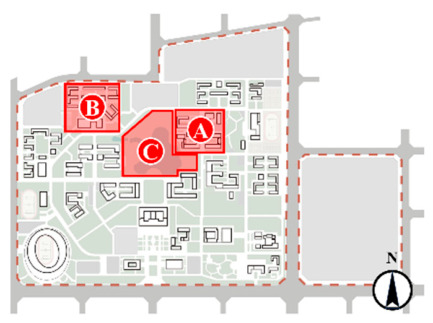
5	Tianjin University—Beiyangyuan Campus (TJU-B)	Region: Tianjin Built in: 2015 Scale: 244 ha Plot ratio: 0.66 Students: 17,000	The campus with mixed functions has a large scale and high built-up area density	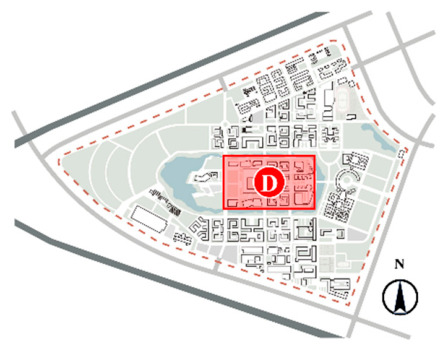

**Table 2:** Campus environmental factors selected for analysis.

Dimension	Topic	Dimension	Topic	Dimension	Topic
Performance	Q30 Outdoor microclimate	Resources	Q45 Pedestrian road safety	Resources	Q60 Ergonomic furniture
	Q31 Outdoor air quality		Q46 Accessibility facilities		Q61 Social venue accessibility
	Q32 Outdoor acoustic		Q47 Landscape accessibility		Q62 Healthy food availability
	Q33 Outdoor lighting		Q48 Landscape diversity		Q63 Healthy food accessibility
	Q34 Outdoor sanitation		Q49 Outdoor landscape maintenance		Q64 Healthy diet promotion
	Q35 Indoor lighting		Q50 Landscape ancillary facilities		Q65 Healthy food supply
	Q36 Indoor thermal		Q51 Environmental safety	Services	Q66 Psychological counseling accessibility
	Q37 Indoor acoustic		Q52 Sports facilities adequacy		Q67 Campus medical services
	Q38 Indoor sanitation		Q53 Sports facilities diversity		Q68 Mental health services
Resources	Q39 Land use efficiency		Q54 Sports facilities accessibility		Q69 Health-related policies
	Q40 Transfer connectivity		Q55 Commercial facilities diversity		Q70 Health education opportunities
	Q41 Pedestrian maintenance		Q56 Commercial facilities accessibility		Q71 Health knowledge promotion
	Q42 Pedestrian width		Q57 Support facilities accessibility	Atmosphere	Q72 Campus activity diversity
	Q43 Pedestrian connectivity		Q58 Indoor landscape visibility		Q73 Course scheduling rationality
	Q44 Pedestrian ancillary facilities		Q59 Stairway/corridor convenience		Q74 Academic support

**Table 3:** Baseline of respondents.

Items		*n*
Distribution	BEAU	90
	NCUT	177
	TJU-W	231
	HUE	216
	TJU-B	189
Gender	Male	342
	Female	561
Grade	Undergraduate	399
	Master student	504
Accommodation	On campus	801
	Off campus	102
Length of life on campus	Less than 1 year	156
	1–2 years	333
	2–3 years	186
	3–4 years	90
	More than 4 years	138
Total		903

**Table 4:** Hierarchical regression results of lifestyle parameters and health status.

	Block 1		Block 2		Block 3		Block 4		Block 4 VIF
	β	*p*	β	*p*	β	*p*	β	*p*	
Campus 2	−0.018	0.72	−0.037	0.404	−0.034	0.442	−0.021	0.625	2.435
Campus 3	−0.079	0.142	−0.088	0.062	−0.086	0.069	−0.078	0.096	2.741
Campus 4	−0.039	0.464	−0.061	0.194	−0.055	0.238	−0.052	0.260	2.651
Campus 5	−0.041	0.431	−0.022	0.628	−0.018	0.683	−0.019	0.671	2.503
Gender	−0.143	<0.001	−0.159	<0.001	−0.160	<0.001	−0.157	<0.001	1.022
Grade	−0.086	0.009	−0.112	<0.001	−0.104	<0.001	−0.107	<0.001	1.029
Accommodation	0.009	0.782	−0.008	0.793	−0.013	0.661	−0.016	0.579	1.021
Length of campus life	0.025	0.455	0.012	0.667	0.013	0.657	0.008	0.765	1.014
Physical exercise frequency	—	—	0.179	<0.001	0.128	0.006	0.115	0.012	2.647
Physical exercise duration	—	—	0.307	<0.001	0.229	<0.001	0.162	0.002	3.542
Active commuting frequency	—	—	0.130	<0.001	0.127	<0.001	0.127	<0.001	1.023
Active commuting duration	—	—	−0.041	0.161	−0.036	0.217	−0.039	0.176	1.025
Nature contact frequency	—	—	—	—	0.147	0.003	0.113	0.023	3.108
Nature contact duration	—	—	—	—	−0.032	0.263	−0.032	0.266	1.023
Social interaction frequency	—	—	—	—	0.036	0.223	0.032	0.270	1.040
Social interaction duration	—	—	—	—	−0.020	0.490	−0.021	0.469	1.039
Healthy diet frequency	—	—	—	—	—	—	0.126	<0.001	1.032
Unhealthy diet frequency	—	—	—	—	—	—	−0.029	0.311	1.019
Health education frequency	—	—	—	—	—	—	0.028	0.323	1.024
Self-rated health literacy	—	—	—	—	—	—	0.140	0.001	2.179

Note: *n* = 903; VIF = variance inflation factor from Model 4. All continuous predictors were mean-centered. Campus 1 (BEAU) served as the reference category.

**Table 5:** Summary table of EFA indexes.

	Category	Eigen Value (Rotated)	Cumulative Variance	Items	Communality	Cronbach α
F1	Pedestrian Infrastructure	3.606	8.014	Q41–46	0.523–0.674	0.833
F2	Outdoor Environmental Quality	3.271	15.282	Q30–34	0.581–0.732	0.863
F3	Landscape Environment	3.173	22.333	Q47–51	0.551–0.688	0.854
F4	Healthy Food Environment	2.738	28.418	Q62–65	0.670–0.697	0.843
F5	Indoor Environmental Quality	2.723	34.469	Q35–38	0.655–0.691	0.835
F6	Daily Service & Social Accessibility	2.557	40.15	Q55–57, 61	0.614–0.688	0.807
F7	Sports Facilities	2.226	45.097	Q52–54	0.725–0.742	0.821
F8	Campus Activities & Academic Support	2.192	49.968	Q72–74	0.719–0.739	0.810
F9	Health Policy & Promotion	2.182	54.818	Q69–71	0.691–0.756	0.808
F10	Medical & Psychological Services	2.181	59.664	Q66–68	0.698–0.757	0.806
F11	Indoor Spatial Quality	2.146	64.434	Q58–60	0.680–0.750	0.795
F12	Land Use & Transit Connectivity	1.656	68.113	Q39–40	0.798–0.828	0.781

Note: *n* = 903; KMO = 0.816; Bartlett’s χ^2^(990) = 15,985.98, *p* < 0.001. The 12 factors collectively explained 68.11% of the total variance. Cronbach’s α was computed from the composite factor scores.

**Table 6:** Environmental factors associated with physical activity parameters.

Factor		Physical Exercise Frequency			Physical Exercise Duration			Active Commuting Frequency		
		β	*p*	LASSO	β	*p*	LASSO	β	*p*	LASSO
F1 Pedestrian Infrastructure		0.211 **	<0.001	0	0.046	0.165	0	0.298 **	<0.001	0.096
F2 Outdoor Environmental Quality		0.277 **	<0.001	0.014	0.163 **	<0.001	0	0.288 **	<0.001	0.07
F3 Landscape Environment		0.095 **	0.003	0	0.046	0.176	0	0.269 **	<0.001	0.057
F4 Healthy Food Environment		−0.030	0.347	0	−0.004	0.898	0	−0.016	0.622	0
F5 Indoor Environmental Quality		0.035	0.280	0	0.029	0.379	0	0.065 *	0.046	0
F6 Daily Service & Social Accessibility		0.079 *	0.011	0	0.040	0.218	0	0.039	0.219	0
F7 Sports Facilities		0.324 **	<0.001	0.074	0.166 **	<0.001	0	−0.016	0.613	0
F8 Campus Activities & Academic Support		0.037	0.231	0	0.311 **	<0.001	0.045	0.120 **	<0.001	0.016
F9 Health Policy & Promotion		0.013	0.692	0	0.133 **	<0.001	0	0.043	0.182	0
F10 Medical & Psychological Services		0.004	0.897	0	0.000	0.991	0	0.063 *	0.047	0
F11 Indoor Spatial Quality		0.046	0.151	0	0.022	0.500	0	0.057	0.078	0
F12 Land Use & Transit Connectivity		0.130 **	<0.001	0	0.031	0.343	0	0.231 **	<0.001	0.048
Hierarchical regression model	R^2^	0.210		-	0.142		-	0.184		-
	Adj-R^2^	0.192		-	0.123		-	0.166		-
	F	11.751		-	7.323		-	9.964		-
	ΔR^2^	0.192		-	0.132		-	0.181		-
	ΔF	17.830		-	11.336		-	16.333		-

Note: *n* = 903, * *p* < 0.05, ** *p* < 0.01.

**Table 7:** Environmental factors associated with Nature contact and communication behavior parameters.

Factor		β	*p*	LASSO
F1 Pedestrian Infrastructure		0.134 **	<0.001	0
F2 Outdoor Environmental Quality		0.096 **	0.005	0
F3 Landscape Environment		0.238 **	<0.001	0.015
F4 Healthy Food Environment		−0.002	0.960	0
F5 Indoor Environmental Quality		0.190 **	<0.001	0
F6 Daily Service & Social Accessibility		−0.002	0.948	0
F7 Sports Facilities		0.001	0.964	0
F8 Campus Activities & Academic Support		0.129 **	<0.001	0
F9 Health Policy & Promotion		0.030	0.372	0
F10 Medical & Psychological Services		0.021	0.520	0
F11 Indoor Spatial Quality		0.211 **	<0.001	0
F12 Land Use & Transit Connectivity		0.027	0.415	0
Hierarchical regression model	R^2^	0.124		-
	Adj-R^2^	0.104		-
	F	6.226		-
	ΔR^2^	0.115		-
	ΔF	9.668		-

Note: *n* = 903, ** *p* < 0.01.

**Table 8:** Environmental factors associated with diet and literacy parameters.

Factor		Healthy Diet Frequency			Self-Rated Health Literacy		
		β	*p*	LASSO	β	*p*	LASSO
F1 Pedestrian Infrastructure		0.037	0.209	0	0.071 *	0.019	0
F2 Outdoor Environmental Quality		0.062 *	0.041	0	0.023	0.468	0
F3 Landscape Environment		−0.007	0.822	0	0.048	0.123	0
F4 Healthy Food Environment		0.573 **	<0.001	0.318	0.167 **	<0.001	0
F5 Indoor Environmental Quality		−0.011	0.715	0	0.063 *	0.042	0
F6 Daily Service & Social Accessibility		0.033	0.241	0	−0.001	0.977	0
F7 Sports Facilities		−0.005	0.856	0	−0.032	0.278	0
F8 Campus Activities & Academic Support		−0.010	0.723	0	0.098 **	0.001	0
F9 Health Policy & Promotion		0.102 **	0.001	0	0.491 **	<0.001	0.171
F10 Medical & Psychological Services		0.054	0.059	0	0.279 **	<0.001	0.036
F11 Indoor Spatial Quality		−0.007	0.802	0	0.040	0.192	0
F12 Land Use & Transit Connectivity		0.034	0.242	0	0.032	0.289	0
Hierarchical regression model	R^2^	0.324		-	0.272		-
	Adj-R^2^	0.308		-	0.256		-
	F	21.106		-	16.483		-
	ΔR^2^	0.31		-	0.26		-
	ΔF	33.711		-	26.268		-

Note: *n* = 903, * *p* < 0.05, ** *p* < 0.01.

**Figure A1:**
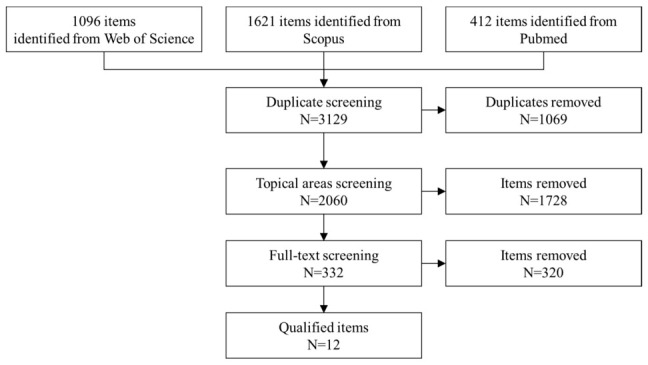
The screening process.

## Data Availability

The data presented in this study are available on request from the corresponding author.

## Abbreviations

The following abbreviations are used in this manuscript:

HEI	Higher Education Institution

## Appendix A

The process of screening environmental assessment tools for healthy campuses consisted of two stages: literature screening and eligibility assessment. As illustrated in Figure A1 and based on the criteria outlined in Table A1, this process resulted in 12 eligible environmental assessment tools for healthy campuses.

**Table A1:** Inclusion criteria for screening.

Screening Phase	Inclusion Criteria
Literature	Campus environment related
	Students’ health related
	Systematic assessment tools are mentioned
Assessment tools	Available in publications and website
	Evidence-based development process
	Validated on campus
	Environment specific tools or integrated tools including environment

The following is a brief description of each assessment tool:

(1) Healthy Campus Standard (HCS)—Developed and administered by the International University Sports Federation (FISU). HCS serves as an assessment tool for the HC in universities worldwide.
(2) Healthy Green School and College (HGSC)—A healthy campus certification standard jointly developed by the Healthy School Campaign and Green Seal, targeting both K–12 schools and higher education institutions.
(3) Healthy University Rating System (HURS)—Developed and operated by the ASEAN University Network–Health Promotion Network (AUN-HPN) with support from the Thai Health Promotion Foundation.
(4) Healthy University Self-Review Tool (HURST)—Developed by the UK Healthy University Network and applied within the network and across multiple Commonwealth universities.
(5) Campus Outdoor Space Evaluation Toolkit (COSE)—Developed and validated by researchers at the University of Guelph, Canada.
(6) College Campus Environment Scale (CCES)—Established by developers at the City University of New York and validated across several institutions.
(7) The Policies, Opportunities, Initiatives, and Notable Topics Audit (POINTS)—Developed and cross-institutionally validated by a collaborative research team from multiple US universities.
(8) College Environmental Perceptions Survey (CEPS)—Developed by the Healthy Campus Research Consortium (HCRC), a multi-state research group under the US Department of Agriculture (USDA).
(9) Campus Assessment Tool (CAT)—Originated from a participatory research project led by youth scholars in Canada, regularly tested across multiple campuses.
(10) Behavior Environment Perception Survey (BEPS)—A campus environment perception measure developed by researchers from the University of Maine, West Virginia University, and others.
(11) University Health Index (UHI)—Developed by researchers at Virginia Tech under the guidance of the US Centers for Disease Control and Prevention, validated using data from the National College Health Assessment.
(12) Physical Activity Campus Environmental Supports Audit (PACES)—Developed with funding from the US Department of Agriculture’s National Institute of Food and Agriculture.

## Appendix B

The questionnaire used in this study is presented in English below.

Dear Respondent,

Hello! We are conducting a research project on healthy campuses in higher education institutions. This questionnaire aims to collect information regarding your health status, lifestyle behaviors on campus, and feedback on various aspects of the university environment. All responses will be anonymized and used solely for the purpose of data analysis in this study. Please feel free to complete the questionnaire. Thank you for your support and cooperation!

**Background Information**

1. Your university and campus: ______

2. Gender: A. Male; B. Female

3. Grade level/status: A. Undergraduate; B. Master’s student; C. Other ______

4. Do you live on campus? A. Yes; B. No; C. Other ______

5. Duration of study/work on campus: A. Less than 1 year; B. 1–2 years; C. 2–3 years; D. 3–4 years; E. More than 4 years

**Health Status**

Please select the option that best describes your health status in the past week.

	Question	Response
6	In general, how would you rate your current health status?	A. Excellent; B. Very good; C. Good; D. Fair; E. Poor
6.1	In the past year, have you had any long-term illnesses?	A. Yes; B. No
6.2	If yes, have these illnesses limited your daily activities?	A. Yes; B. No
7	Moderate activities, such as moving a table, cleaning the floor, bowling, or practicing physical exercises:	A. Limited a lot; B. Limited a little; C. Not limited at all
8	Does your health limit you in walking upstairs?	A. Limited a lot; B. Limited a little; C. Not limited at all
6	In general, how would you rate your current health status? (Repeated check)	A. Excellent; B. Very good; C. Good; D. Fair; E. Poor
9	Do you feel less capable than usual in daily life or work due to physical health reasons?	A. Yes; B. No
10	In your work or daily activities, are you limited in what you do due to physical health reasons?	A. Yes; B. No
11	Do you feel less capable than usual in your work or daily activities due to emotional problems (e.g., feeling depressed or anxious)?	A. Yes; B. No
12	In your work or daily activities, are you limited in what you do due to emotional problems?	A. Yes; B. No
13	How much do physical discomforts such as fatigue or exhaustion affect your daily work?	A. Not at all; B. Slightly; C. Moderately; D. Quite a lot; E. Extremely; F. Not applicable
14	How much of the time have you felt calm and peaceful?	A. All of the time; B. Most of the time; C. A good bit of the time; D. Some of the time; E. A little of the time; F. None of the time
15	How much of the time have you felt full of energy?	A. All of the time; B. Most of the time; C. A good bit of the time; D. Some of the time; E. A little of the time; F. None of the time
16	How much of the time have you felt downhearted, blue, or depressed?	A. All of the time; B. Most of the time; C. A good bit of the time; D. Some of the time; E. A little of the time; F. None of the time
17	How much of the time has your physical health or emotional problems interfered with your social activities?	A. All of the time; B. Most of the time; C. A good bit of the time; D. Some of the time; E. A little of the time; F. None of the time

**Lifestyle on Campus**

Please select the option that best describes your usual lifestyle on campus.

	Statement	Response
18	I frequently engage in physical exercise.	1–5
19	I spend a lot of time on physical exercise.	1–5
20	I often walk or cycle on campus for commuting.	1–5
21	I spend a lot of time walking or cycling on campus.	1–5
22	I frequently have contact with natural environments.	1–5
23	I spend a lot of time in contact with natural environments.	1–5
19	I spend a lot of time on physical exercise. (Repeated check)	1–5
24	I often interact with others on campus.	1–5
25	I spend a lot of time interacting with others.	1–5
26	I often choose foods such as vegetables, fruits, and dairy products.	1–5
27	I often choose fast food, fried foods, and similar items.	1–5
28	I frequently participate in health-related courses or training on campus.	1–5
29	I feel that I have a good understanding of health-related knowledge.	1–5

**Campus Environment**

Please select the option that best describes your campus environment.

	Question	Response
30	I find the outdoor temperature and wind speed on campus pleasant.	1–5
31	I feel that the outdoor air on campus is fresh.	1–5
32	I am rarely bothered by noise when outdoors on campus.	1–5
33	The outdoor lighting on campus is adequate at night.	1–5
34	I feel that the outdoor areas on campus are clean.	1–5
35	The indoor lighting on campus is adequate.	1–5
36	I find the indoor temperature comfortable.	1–5
37	I am rarely bothered by noise when indoors.	1–5
38	The indoor areas of campus buildings are clean.	1–5
39	Land on campus is fully utilized, with little idle land.	1–5
40	The connection between off-campus public transportation and the campus is convenient.	1–5
41	Pedestrian and bicycle paths on campus are free from obstacles and damage that would affect my travel.	1–5
42	I rarely have to walk on other lanes due to insufficient width of pedestrian or bicycle paths.	1–5
43	Pedestrian and bicycle paths on campus are continuous, separated, and meet my travel needs.	1–5
44	There are often shaded areas, seats, or small gardens near pedestrian paths for me to rest.	1–5
45	I feel safe from motor vehicles or other traffic when walking or cycling.	1–5
46	Accessible facilities are available on pathways to various places on campus.	1–5
47	I am satisfied with the distance to gardens, green spaces, plazas, lakes, and other landscapes on campus.	1–5
48	I feel that the types of landscapes on campus are diverse.	1–5
49	The outdoor landscapes on campus are aesthetically pleasing and well-maintained.	1–5
50	There are often convenient facilities such as shaded areas and seats near campus landscapes.	1–5
51	I feel safe when engaging in activities on campus.	1–5
52	There are sufficient sports facilities on campus, and I rarely miss out on exercise due to a lack of facilities.	1–5
53	The variety of fitness facilities on campus meets my exercise preferences.	1–5
54	I am satisfied with the walking distance to sports facilities and venues on campus.	1–5
55	There is a variety of daily service facilities on campus that meet my needs.	1–5
56	I am satisfied with the walking distance to supermarkets, shops, and other commercial facilities on campus.	1–5
57	I am satisfied with the walking distance to printing, laundry, and other support facilities on campus.	1–5
58	I can often find plants indoors or see trees and grass through the windows.	1–5
59	The stairs and corridors of common buildings on campus are easy to use.	1–5
60	The seats in classrooms, offices, and laboratories are comfortable to use.	1–5
61	There are often places on campus where I can talk with others.	1–5
62	There are many places on campus that sell milk, fruit, and similar foods.	1–5
63	I am satisfied with the distance to places on campus that sell milk, fruit, and similar foods.	1–5
64	There is a lot of information on campus promoting healthy eating.	1–5
65	Campus dining facilities provide adequate healthy foods such as milk, fruits, and grains, at prices that do not cause me concern.	1–5
66	I am satisfied with the distance to the campus health center and psychological counseling center.	1–5
67	I am satisfied with the medical services provided on campus.	1–5
68	I am satisfied with the mental health services provided on campus.	1–5
69	The university has established a variety of health-related policies.	1–5
35	The indoor lighting on campus is adequate. (Repeated check)	
70	There are ample opportunities on campus for me to acquire health knowledge and sports skills.	1–5
71	There is a lot of information on campus promoting health knowledge.	1–5
72	There is a wide variety of activities on campus.	1–5
73	The class schedule on campus is reasonable, and there are few instances of continuous classes throughout the day.	1–5
74	I can always easily access academic support on campus.	1–5

## Appendix C

The exploratory factor analysis results of 45 questionnaire variables are as follows.

**Table A2:** The KMO Bartlett test (*n* = 903).

Test	Value
Kaiser–Meyer–Olkin Measure of Sampling Adequacy	0.816
Bartlett’s Test of Sphericity—Approx. Chi-Square	15,985.977
df	990
*p*	<0.001

**Table A3:** The variance of EFA explained (*n* = 903).

Factor	Initial Eigenvalue	Initial (%)	Initial Cum (%)	Rotated SSL	Rotated (%)	Rotated Cum (%)
1	5.19	11.534	11.534	3.606	8.014	8.014
2	3.454	7.675	19.209	3.271	7.268	15.282
3	3.386	7.525	26.733	3.173	7.051	22.333
4	3.113	6.917	33.651	2.738	6.085	28.418
5	2.623	5.828	39.479	2.723	6.051	34.469
6	2.404	5.342	44.82	2.557	5.681	40.15
7	2.27	5.045	49.866	2.226	4.947	45.097
8	2.004	4.454	54.32	2.192	4.872	49.968
9	1.752	3.894	58.214	2.182	4.849	54.818
10	1.626	3.613	61.827	2.181	4.847	59.664
11	1.454	3.23	65.057	2.146	4.77	64.434
12	1.411	3.137	68.193	1.656	3.679	68.113

**Table A4:** The factor loadings (*n* = 903).

Itmes	F1	F2	F3	F4	F5	F6	F7	F8	F9	F10	F11	F12	Commonality
Q30	0.024	0.759	0.003	−0.013	−0.042	0.006	−0.003	−0.022	0.023	0.037	0.004	−0.016	0.582
Q31	−0.016	0.85	0	−0.001	−0.051	0.061	−0.035	0.017	−0.024	0.005	−0.013	−0.017	0.732
Q32	−0.021	0.809	0.018	0.03	−0.014	−0.018	0.007	0.023	0.003	−0.024	0.039	0.009	0.66
Q33	0.007	0.745	0.014	0.016	0.134	−0.049	−0.012	0.02	−0.044	−0.001	0.019	0.045	0.581
Q34	0.005	0.844	−0.031	−0.03	−0.007	−0.008	0.054	−0.021	0.03	−0.03	−0.047	−0.004	0.722
Q35	0.011	−0.025	0.003	−0.01	0.815	0.01	−0.014	0.036	0.012	−0.03	0.031	−0.003	0.669
Q36	−0.035	0	−0.01	0.019	0.806	−0.012	−0.009	−0.039	0.019	−0.009	0.037	0.002	0.655
Q37	0.038	0.035	0.015	0.028	0.818	−0.005	0.033	0.002	−0.05	0.037	−0.064	−0.055	0.685
Q38	−0.023	−0.014	−0.003	−0.046	0.827	0.027	−0.018	−0.015	0.039	−0.006	−0.005	0.037	0.691
Q39	0.017	0.027	−0.012	0.028	0.013	−0.029	−0.008	−0.007	−0.02	0.024	−0.009	0.891	0.798
Q40	−0.007	−0.015	0.031	−0.025	−0.034	0.023	−0.009	0.003	0.015	−0.024	0.012	0.908	0.828
Q41	0.739	−0.052	−0.051	−0.006	0.015	0.013	0.039	0.044	0.002	−0.014	−0.025	0.097	0.565
Q42	0.814	0.004	−0.016	0.044	−0.052	−0.003	−0.031	−0.025	0.033	−0.03	0.054	−0.026	0.674
Q43	0.771	−0.004	0.03	−0.015	0.023	0.019	−0.011	−0.023	−0.048	0.033	0.016	−0.003	0.602
Q44	0.718	0.051	0.007	0.03	0.001	−0.01	0.006	−0.003	0.028	0.033	−0.039	0.005	0.523
Q45	0.804	0.017	0.013	−0.027	0.019	−0.014	−0.051	0.022	0.019	−0.025	0.003	−0.03	0.653
Q46	0.787	−0.016	0.023	−0.03	−0.015	−0.007	0.044	−0.025	−0.029	−0.005	−0.003	−0.036	0.626
Q47	0.047	−0.105	0.82	−0.006	0.031	0	−0.015	0.019	−0.024	−0.014	0.024	0.003	0.688
Q48	−0.034	0.051	0.817	−0.004	−0.034	0.034	0.005	−0.047	0.04	−0.019	−0.003	0.02	0.678
Q49	0.001	0.091	0.772	0.007	0.02	0	−0.048	0.011	−0.023	0.015	0.011	−0.035	0.608
Q50	−0.009	−0.036	0.739	−0.013	0	−0.023	0.036	0.032	−0.015	−0.001	−0.005	0.02	0.551
Q51	−0.001	0.022	0.812	0.011	−0.013	0.004	0.024	−0.017	0.03	0.025	−0.027	0.005	0.663
Q52	0.023	0.027	−0.006	−0.006	0.008	0.016	0.849	0.009	−0.02	0.021	−0.014	−0.035	0.725
Q53	−0.022	−0.007	0.018	0.004	−0.003	−0.036	0.86	0.004	0.02	−0.018	0.008	−0.006	0.742
Q54	−0.003	−0.007	−0.002	−0.006	−0.013	0.03	0.856	−0.009	0.012	−0.009	0.021	0.024	0.735
Q55	−0.025	−0.017	−0.007	0.028	0.044	0.791	0.05	−0.044	−0.033	0.047	−0.018	0.063	0.642
Q56	−0.002	0.005	0.033	−0.017	−0.006	0.827	−0.028	−0.026	0.001	−0.013	0.005	−0.019	0.688
Q57	0.024	0.001	−0.035	0.033	0.021	0.782	0.03	0.054	−0.023	0.023	0.02	−0.014	0.62
Q58	−0.015	−0.03	0.101	0.065	−0.01	−0.046	0.025	−0.003	−0.005	−0.004	0.813	−0.009	0.68
Q59	−0.009	0	−0.062	−0.021	0.092	0.012	−0.004	0.009	0.007	0	0.847	0.022	0.732
Q60	0.025	0.029	−0.039	−0.041	−0.073	0.034	−0.005	−0.006	−0.005	0.011	0.859	−0.008	0.75
Q61	0.001	0.009	0.018	−0.029	−0.037	0.776	−0.04	0.019	0.046	−0.057	−0.005	−0.032	0.614
Q62	−0.018	−0.052	0.034	0.83	0.012	0.009	0.002	0.007	0.009	−0.039	−0.038	0.023	0.697
Q63	0.005	−0.024	0.021	0.827	0.002	0.004	−0.028	0.058	−0.014	0.036	−0.003	−0.027	0.691
Q64	−0.005	0.047	−0.003	0.822	−0.027	−0.008	0.007	−0.069	0.008	−0.031	−0.013	0.003	0.684
Q65	0.013	0.027	−0.06	0.813	0.004	0.008	0.011	0.004	0.005	0.028	0.054	0.002	0.67
Q66	0.008	0.045	−0.015	−0.038	−0.011	0.074	−0.002	−0.017	0.007	0.864	0.013	0.035	0.757
Q67	0.02	−0.039	0.012	0.048	−0.007	0.007	−0.027	0.019	0.045	0.831	−0.012	−0.005	0.698
Q68	−0.032	−0.017	0.008	−0.017	0.01	−0.082	0.022	−0.002	−0.035	0.849	0.007	−0.03	0.731
Q69	−0.059	−0.006	−0.008	−0.006	−0.043	0.016	−0.019	−0.003	0.851	0.032	−0.025	0.007	0.731
Q70	0.043	0.02	−0.019	0.016	0.062	−0.037	0.035	0.034	0.824	0.005	−0.014	0.038	0.691
Q71	0.024	−0.017	0.03	−0.001	0.003	0.014	−0.002	−0.025	0.866	−0.023	0.035	−0.048	0.756
Q72	−0.01	−0.036	0.003	0.024	−0.013	−0.007	0.057	0.848	−0.038	−0.007	−0.029	0.017	0.726
Q73	−0.031	0.025	0.009	−0.05	−0.01	0.007	−0.033	0.842	0.047	0.005	0.044	0.016	0.719
Q74	0.031	0.025	−0.007	0.022	0.009	0.005	−0.019	0.857	−0.004	0.001	−0.016	−0.036	0.739
